# Characterization of Primary Standards for Use in the HPLC Analysis of the Procyanidin Content of Cocoa and Chocolate Containing Products

**DOI:** 10.3390/molecules14104136

**Published:** 2009-10-15

**Authors:** William J. Hurst, Bruce Stanley, Jan A. Glinski, Matthew Davey, Mark J. Payne, David A. Stuart

**Affiliations:** 1 The Hershey Center of Health and Nutrition, The Hershey Company, 1025 Reese Avenue, Hershey, PA 17033, USA; 2 Core Proteomics Facility, MS Hershey Medical Center, Hershey, PA 17033, USA; 3 Planta Analytica, 39 Rose Ave, Danbury, CT 06810, USA

**Keywords:** procyanidin, cocoa, chocolate, flavanols, HPLC, MALDI, reference standards, mass spectrometry

## Abstract

This report describes the characterization of a series of commercially available procyanidin standards ranging from dimers DP = 2 to decamers DP = 10 for the determination of procyanidins from cocoa and chocolate. Using a combination of HPLC with fluorescence detection and MALDI-TOF mass spectrometry, the purity of each standard was determined and these data were used to determine relative response factors. These response factors were compared with other response factors obtained from published methods. Data comparing the procyanidin analysis of a commercially available US dark chocolate calculated using each of the calibration methods indicates divergent results and demonstrate that previous methods may significantly underreport the procyanidins in cocoa-containing products. These results have far reaching implications because the previous calibration methods have been used to develop data for a variety of scientific reports, including food databases and clinical studies.

## Introduction

**Figure 1:**
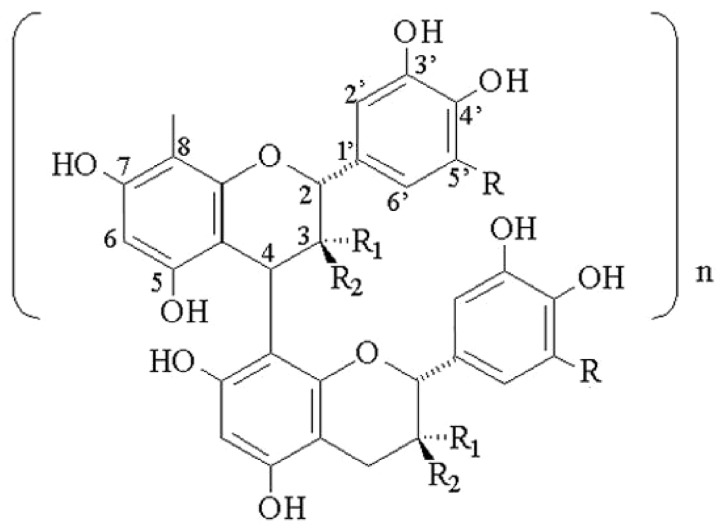
Chemical structure of proanthocyanidins. Where R = H, it is a procyanidin: catechin (R_1_ = H and R_2_ = OH) and epicatechin (R_1_ = OH and R_2_ = H).

Procyanidins or condensed tannins, are oligomeric compounds comprised of catechin and epicatechin, joined primarily via 4-8 and 2-7 linkages. Other linkages are less common, but nevertheless they contribute to the multiplicities of discrete species comprising families of dimeric (DP = 2), trimeric (DP = 3) and higher DPs. These compounds are ubiquitous in Nature and can be subdivided into type A and type B, with this division being based on the linkage among the various monomeric units. An example of the B-type linkage, which is common in cocoa, grapes and wine, is shown in Figure 1. Their physical makeup ranges from dimers through polymers with polymers ranging from 3,000 amu and higher [1,2,3]. Before current HPLC methods were developed, methods to measure members of this class were based on colorimetric reactions or thin layer chromatography (TLC) [4]. The complexity of oligomeric composition is reflected in analytical chemistry by a difficulty in chromatographic separation. While reverse phase chromatography can resolve individual oligomers below DP = 5 relatively well, normal phase can be used to separate oligomeric procyanidins according to their DP. Methods have been designed to achieve separation of oligomers above DP = 5. In this modality, the broadness of eluting bands representing given DP stems from the heterogenicity of the band, e.g. the dimeric band may represent up to eight different dimers, while the octamer band may represent hundreds of individual species. As these compounds are members of the larger class of polyphenols , whose number exceeds 5,000, the interest in them is obvious.

There are those who report physiological action from procyanidins, while others indicate they serve as a substrate for catechin and epicatechin, but that discussion is beyond the scope of this report [5]. As analytical methods evolve, so do methods for the determination of natural products, including procyanidins. Over the past decade there have been a number of HPLC methods published on the determination of procyanidins in foodstuffs, including apples, cranberries, blueberries, and cocoa [6,7,8,9,10]. These HPLC methods have involved a number of separation mechanisms including normal phase, reversed phase and size exclusion [11,12]. A normal phase method initially based on a silica column and now using a diol column with fluorescence detection has become almost a *de facto* standard method used by many commercial and industrial laboratories [13]. One of the concerns surrounding this method is the choice of an appropriate standard material, since some authors in earlier studies reported on the use of epicatechin as a standard, while others have reported data on lab-produced standards which are not available in ordinary commerce. Additionally there is the complicating fact that oligomeric flavanols suffer from severe fluorescence quenching, making quantification of oligomers or polymers difficult or impossible without the use of appropriate standards [14].

This paper reports on the characterization of commercially available standards from DP = 2 through DP = 10 and their application to the determination of procyanidins through decamers in cocoa. We also compare these results to published response factor data for this assay and discuss the implications of applying a new set of standards for the reporting of procyanidins in cocoa-containing foods and other natural products.

## Results

**Figure 2:**
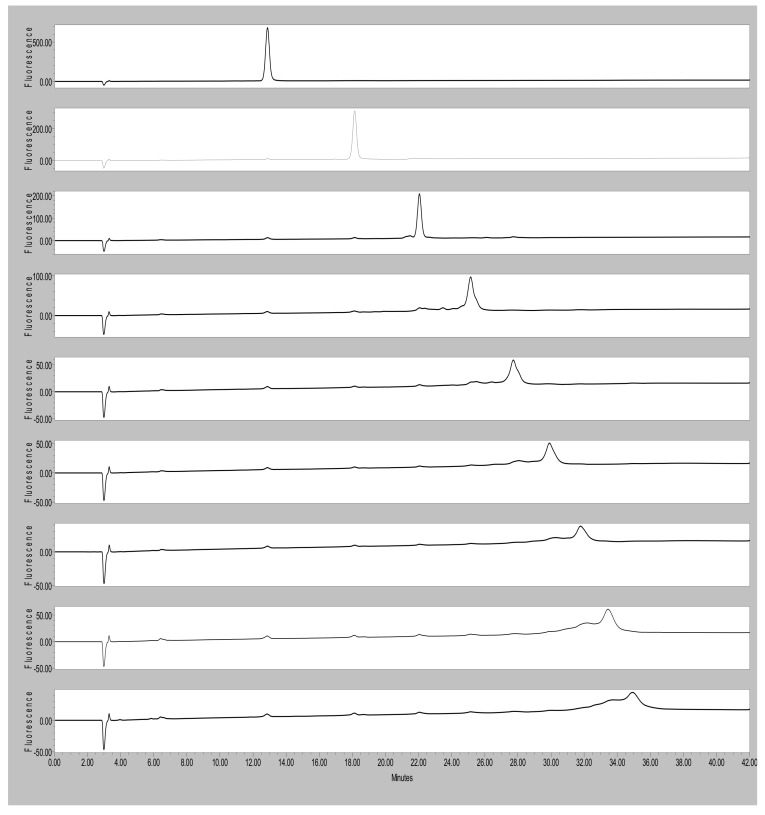
Top to bottom: HPLC-FL separation of DP2 through DP10 procyanidin standards.

**Figure 3:**
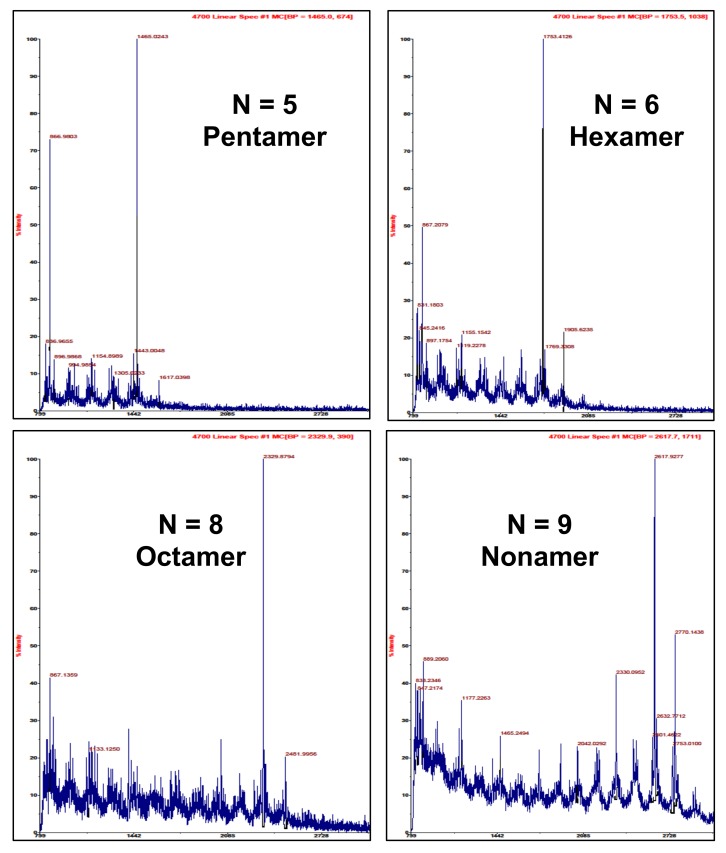
MALDI mass spectra of selected cocoa procyanidin standards.

The comparison of the HPLC with fluorescent detection of the isolated cocoa procyanidins is shown in Figure 2 for the DP = 2 through DP = 10 oligomers. The DP 2 through 7 oligomers show excellent purity by HPLC. Oligomers 8 through 10 show increasing possible contamination with adjacent oligomers, especially from lower molecular weight species. Selected MALDI mass spectra for oligomers 5, 6, 8, and 9 are shown in Figure 3. The supporting matrix contributes a repeating 154 molecular weight pattern as seen for all the MALDI mass spectra shown. Analysis of the pentamer shows a strong major peak at 1,465 with no larger or smaller peaks with multiples of 288 molecular weight, which would be indicative of shorter or longer oligomers, thus indicating excellent purity. Results for the hexamer are essentially the same, except the main peak is at 1,753. The MALDI mass spectra for the octamer shows a major peak at 2,329 with no larger peaks, but a smaller peak at about 2,041 possibly indicating some heptamer contamination. Results for the nonamer show a major peak at 2,618 and a minor smaller peak at 2,030, possibly indicating some octamer contamination. A summary of the major peaks for the isolated cocoa procyanidin oligomers is shown in Table 1 with the mass that is given being that of the sodium adduct. Additionally, an examination of the mass spectra does not indicate the presence of type A procyanidins, which would have been evidenced by a 2 amu difference [15,16].

To use these isolated cocoa standards for measuring the level of procyanidins in cocoa or chocolate, we have estimated the purity of each preparation based on review of HPLC and MALDI information and these estimates are shown in Table 2. Some recommend the use of NMR to develop additional data on members of this compound class but the complexity of chemical shift information can make the interpretation of resulting spectra problematic. 

**Figure 4:**
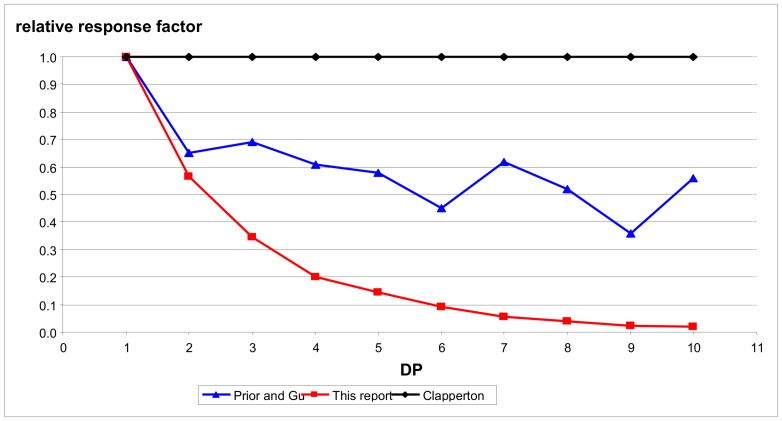
Relative Response Factors (RRF) for Three Standard Types.

While MS alone does not provide information on stereochemistry, the combination of HPLC with fluorescence detection and MALDI MS provides substantial evidence to attest to the purity of these standards. With these estimated purities for the isolated procyanidins, calculations were made of the relative fluorescence response factor (RRF) expected for each of the cocoa oligomers and results are shown in Table 3. Also shown are the published relative response factors of Clapperton *et al.* [9] and Prior and Gu [5]. In the case of Clapperton, he reported on the use of epicatchin as a standard, therefore 1.0 was used as a response factor for degrees of polymerization from 1 to 10. The response factors described here based on the isolated and purity adjusted procyanidin fractions decrease in a predictable manner as oligomer chain length increases. The previously published response factors do not show such a relationship. The results of this comparison are shown in Figure 4.

In Table 4 is shown an example of the repeated analysis of a commercially available dark chocolate using the newly characterized standard materials and a comparison of the results with other published response factors. The results demonstrate that the use of the standards described here result in a 71% to 108% higher determination of the total level of flavanols 1 through 10 for the same dark chocolate. In examining individual oligomers such as the heptamer (DP = 7), for example, one can see that there is an increasing difference in the estimation, with at least an 18-fold higher level found using the current standards compared to using response factors published earlier [5,9]. 

## Discussion

The results described here characterize the first commercially available cocoa procyanidin standards. Our results show that these standards have excellent purity, especially for DP - 2 through 7, making them suitable for use as a primary standard for procyanidin determination. While the HPLC (Figure 2) and the MALDI MS (Figure 3) data is useful in establishing the purity of these materials as a primary standard for the procyanidin assay for cocoa and chocolate products, the potential implications of such standards are wide reaching. Much of the data that has been published or is in databases regarding procyanidin content of foodstuffs is based on the use of epicatechin alone as a standard or the use of standards not commercially available [5,9]. The data shown in Table 3 suggest that use of either of these methods can result in substantial underreporting of the procyanidin content of these products. Figure 4 provides an illustration of response factors developed using several of these methods. These data indicate that the previously published results reported for chocolate and cocoa using the HPLC fluorescence method may have dramatically underreported the procyanidin content of these raw materials and products. 

The studies reported here were limited to oligomers two through ten for a number of reasons. First, the standards used here have diminishing purity at the highest DP levels. There also is concern about what should be used as a standard for cocoa polymers, which have a reported average DP for polymers of 13 [13]. Published reports that have estimated food procyanidin polymers have used blueberry polymers, which have a DP= 36, for standards. It is likely that only a true cocoa derived procyanidin standard will suffice as a standard. Finally, because the relative response factors for chromatographic peaks require such large correction, it is likely that attempting to quantify larger peaks using fluorescence detection will have diminishing reliability for DPs in excess of 10. 

## Experimental

Cocoa reference standards for DP = 2 through DP = 10 were acquired from Planta Analytical and analyzed as is by normal phase HPLC with fluorescence detection to assess purity and also by MALDI-TOF Mass Spectrometry (MS) to develop structural information. Standards for MALDI-TOF MS were dissolved at a concentration of 2 mg/mL in methanol.

### HPLC

A Waters Acquity UPLC system equipped with a diol-based column (Phenomenex, 5 μm, 250 mm × 4.6 mm) at 30 °C and fluorescence detection (276 nm excitation, 316 nm emission) was used. A binary mobile phase was employed consisting of 98:2 CH_3_CN/AcOH and 95:3:2 MeOH/H_2_O/AcOH. The flow rate was 1.0 mL/min and injection volumes were 10 µL. The gradient program is displayed in Table 5. Procyanidin standard concentrations were approximately 10 ppm. Purity was based on the injection of standards prepared at three levels. Standards were prepared at ½X, 1X and 10X concentration used for working standard HPLC calibration. The standard chromatograms were reviewed and all peaks present are integrated. The area % average of six injections for peak of interest was calculated. The primary peak of interest is evaluated as a percent of all peaks from that particular standard, which then is deemed the purity percentage for the neat standard.

### MALDI-TOF mass spectrometry

Duplicate spots of 2 µL of each standard mixture were spotted onto defined locations on a stainless steel MALDI target plate, air-dried, and then overlaid with 0.6 µL of DHB matrix (dihydrobenzoic acid, 20 mg/mL in water). An additional calibration spot containing diluted 4700 Calibration Mix (Applied Biosystems, Framingham, MA, USA; diluted 1:24 with DHB matrix solution) was applied immediately adjacent to the sample spots, as well as an additional spot containing only the DHB matrix. A new linear positive ion mode MS calibration for the Applied Biosystems 4800 MALDI TOF-TOF mass spectrometer was created by averaging 500 laser shots to create an MS spectrum of the five peak masses in the calibration standard spot, and this calibration was used to calibrate all sample spectra from the neighboring spots, which were acquired immediately afterwards.

Each sample spectrum was acquired in linear positive ion mode, with laser power set at 6000 for standards thru DP = 8, and for the higher polymers in standards DP = 9 and DP = 10 requiring additional laser power to ionize sufficiently, the laser power was set to 6,500 and 7,000, respectively). Spectra for each sample were obtained by averaging the spectra from 500 separate laser shots. A CHCA matrix (α-cyanohydroxycinnamic acid, 5 mg/mL in 50% acetonitrile, 0.1% TFA) was also tested (results not shown), but the spectra were inferior to those with the DHB matrix and all subsequent work was done with the DHB matrix.

### Sample Chocolate

The commercially available semi-sweet dark chocolate used in the study to compare response factors was from a single lot of chocolate and was analyzed 14 separate times by the methods previously described [13]. Results were averaged and the standard deviation of the data was calculated. 

## Conclusions

In conclusion, to quantify cocoa procyanidins accurately by the most popular HPLC method, researchers should migrate to the use of commercially available and characterized primary standards such as described in this report. With such standards, one can in turn calibrate secondary standards for routine use. This should result in more accurate data and a reproducible method for the HPLC determination of this class of compounds These results indicate that, for a variety of reasons, currently it is difficult to quantify procyanidins greater that DP = 10 without changes to the separation and detection methodology.

## Figures and Tables

**Table 1:** Summary of the MALDI MS ions for the cocoa procyanidins DP = 2 through 10.

Cocoa Procyanidin Oligomers	m/z ^a^
**Dimer; DP = 2**	601
**Trimer; DP = 3**	889
**Tetramer; DP = 4**	1,177
**Pentamer; DP = 5**	1,465
**Hexamer; DP = 6**	1,753
**Heptamer; DP = 7**	2,041
**Octamer; DP = 8**	2,329
**Nonamer; DP = 9**	2,617
**Decamer; DP = 10**	2,907

^a^ Ion given is sodium adduct.

**Table 2:** Estimation of the purity of each of the isolated cocoa procyanidin preparations.

Cocoa Procyanidin Oligomers	Estimated Purity (%)
**Dimer; DP = 2**	96
**Trimer; DP = 3**	88
**Tetramer; DP = 4**	85
**Pentamer; DP = 5**	70
**Hexamer; DP = 6**	70
**Heptamer; DP = 7**	70
**Octamer; DP = 8**	65
**Nonamer; DP = 9**	60
**Decamer; DP = 10**	50

**Table 3:** Chromatographic response factors for quantification.

Cocoa Oligomer	Standard described in this manuscript	Prior and Gu [5]	Clapperton *et al*. [9]
**Monomer; DP = 1**	1.00	1.00	1.00
**Dimer; DP = 2**	0.57	0.65	1.00
**Trimer; DP = 3**	0.35	0.69	1.00
**Tetramer; DP = 4**	0.20	0.61	1.00
**Pentamer; DP = 5**	0.15	0.58	1.00
**Hexamer; DP = 6**	0.09	0.45	1.00
**Heptamer; DP = 7**	0.06	0.62	1.00
**Octamer; DP = 8**	0.04	0.52	1.00
**Nonamer; DP = 9**	0.03	0.36	1.00
**Decamer; DP = 10**	0.02	0.56	1.00

**Table 4:** Calculation of the procyanidin content of a commercial US dark chocolate in mg/g of product using the response factors (RF) shown in Table 4. Average ± standard deviation reported for RF of this report are for 14 independent determinations of the same production lot of commercial dark chocolate.

Cocoa Oligomer	RF of this Manuscript	RF of Prior and Gu [5]	RF of Clapperton *et al*. [9]
**Monomer**	0.50 ± 0.029	0.50	0.50
**DP = 2**	0.27 ± 0.022	0.20	0.133
**DP = 3**	0.18 ± 0.015	0.084	0.056
**DP = 4**	0.26 ± 0.035	0.063	0.042
**DP = 5**	0.18 ± 0.037	0.049	0.028
**DP = 6**	0.19 ± 0.074	0.042	0.021
**DP = 7**	0.13 ± 0.017	0.007	0.0063
**DP = 8**	0.09 ± 0.015	0.0035	0.0021
**DP = 9**	0.07 ± 0.018	0.0035	0.0014
**DP = 10**	0.07 ± 0.029	0.0021	0.0007
**TOTAL**	**1.66**	**0.954**	**0.788**

**Table 5:** HPLC gradient conditions.

	Time, min	flow	%A	%B	Curve
1		1.00	100.0	0.0	
2	35.00	1.00	60.0	40.0	6
3	39.00	1.00	60.0	40.0	6
4	41.00	1.00	0.0	100.0	6
5	47.00	1.00	0.0	100.0	6
6	51.00	1.00	100.0	0.0	6

A= 98:2 CH_3_CN/acetic acid; B= 95:3:2 MeOH/ H_2_O/acetic acid; Curve 6 is Linear.
